# Parasite species co-occurrence patterns on North American red squirrels (*Tamiasciurus hudsonicus*)

**DOI:** 10.1017/S0031182024001513

**Published:** 2024-12

**Authors:** Jasmine S. M. Veitch, Jeff Bowman, J. Dawson Ketchen, Albrecht I. Schulte-Hostedde

**Affiliations:** 1 Department of Biology, Laurentian University, Sudbury, Ontario, Canada; 2 Faculty of Veterinary Medicine, University of Calgary, Calgary, Alberta, Canada; 3 Ontario Ministry of Natural Resources and Forestry, Wildlife Research and Monitoring Section, Trent University, Peterborough, Ontario, Canada

**Keywords:** community structure, ectoparasites, parasite communities

## Abstract

Parasite species interactions, host biology traits, and external environmental factors can drive co-occurrence patterns between parasite species. We investigated co-occurrence patterns between three ectoparasite species (mite (*Neotrombicula harperi*), and fleas (*Orchopeas caedens* and *Ceratophyllus vison*)) of North American red squirrels (*Tamiasciurus hudsonicus*). We evaluated (1) whether ectoparasites of red squirrels exhibit non-random co-occurrence patterns, and (2) the contribution of host and external environmental factors to parasite co-occurrence. Bayesian ordination and regression analysis (boral) revealed random associations between parasite species pairs when accounting for host and external environmental factors. However, the mite *N. harperi* exhibited a negative association with the flea *O. caedens* and positive association with the flea *C. vison* linked to temporal patterns of occurrence. Our data suggests that parasites of the investigated population of red squirrels tend to form associations based on temporal trends in infestation rather than species interactions. Further experimentation should investigate the role of additional factors on parasite co-occurrence patterns, such as temperature, precipitation, and humidity.

## Graphical abstract

**Figure:**
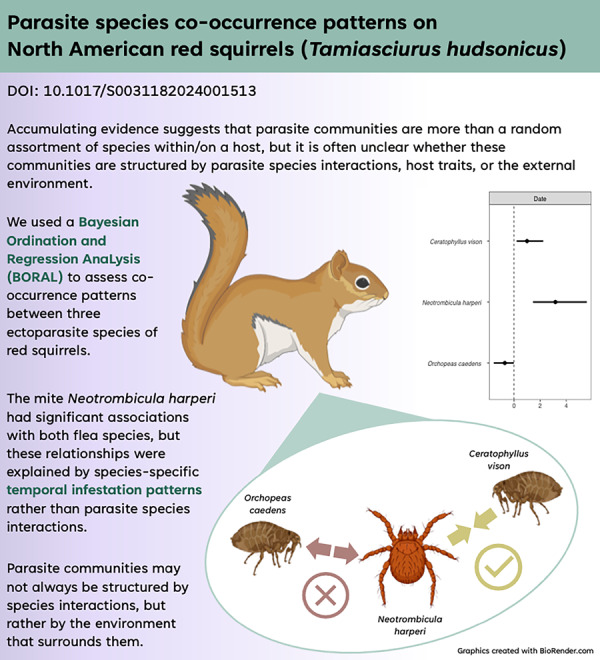

## Introduction

Host-parasite systems provide effective models in community ecology as they allow for replicated units through individual hosts or populations and precise measurements of total species richness (Poulin, 2019). Within a sample of hosts, parasites may exhibit a range of interactions varying from antagonistic to facilitative (Benesh and Kalbe, 2016; Hoffmann *et al*., 2016). Non-random co-occurrence patterns may not only form from species interactions, but also due to responses to environmental conditions. The environment of a parasite includes both the host environment (i.e. host characteristics) and external environment (i.e. host population abundance and habitat of the host; Mouillot *et al*., 2005; Van den Wyngaert *et al*., 2014; Dallas *et al*., 2019). Given that hosts are the habitat for parasites, host traits can alter parasite co-occurrence patterns through habitat preferences along with other host-specific characteristics like immune response (Dallas *et al*., 2019). Additionally, as ectoparasites are in direct contact with both their host and the external environment, abiotic factors can also play a role in structuring ectoparasite communities (Poisot *et al*., 2017). The recent use of joint species distribution modelling (JSDM) allows for the evaluation of associations between parasite species while identifying the role of host traits and external environmental factors (Dallas *et al*., 2019). Therefore, this approach enables the identification of whether co-occurrence patterns between parasites are non-random and whether species associations are shaped by host or external environmental factors can be identified.

Parasite communities can be highly dynamic, with parasite co-occurrence patterns varying over time and space (Krasnov *et al*., 2020). Uneven distributions of parasite species are especially common and can have important ecological consequences on host mortality and immune response, as well as parasite competition and reproduction (McVinish and Lester, 2020). Parasite aggregation is a common ecological pattern that has been observed in many species, including small mammals (Buchholz and Dick, 2017; Krasnov *et al*., 2019; Herrero-Cófreces *et al*., 2021). An aggregated pattern is one whereby many hosts are infected with few or no parasites, but some hosts are infected with many species of parasites. Aggregation generally decreases heterospecific co-occurrences; however, parasite species may influence individual host fitness during parasitic encounters, leading to outcomes other than aggregation (Morrill *et al*., 2017). Parasite communities remain largely understudied compared to free-living communities (Hoffmann *et al*., 2016; Dallas *et al*., 2019). Further investigation of parasite co-occurrence patterns is required to gain insight into community dynamics of parasite assemblages and whether ectoparasites of red squirrels conform to the patterns found in similar host-parasite systems (including aggregation).

North American red squirrels (*Tamiasciurus hudsonicus*) play critical roles in their ecosystems as ecosystem modifiers for mammalian predators (Posthumus *et al*., 2015), important nest and seed predators (Pelech *et al*., 2010; Steele and Yi, 2020), and carriers of zoonotic pathogens (Bangari *et al*., 2007; Stenger *et al*., 2015). Ectoparasites of red squirrels have received attention, primarily around their impact on host reproduction and general infestation patterns. For instance, fleas and mites of red squirrels are known to influence reproductive success, though the direction of this relationship can vary (Gooderham and Schulte-Hostedde, 2011; Patterson *et al*., 2013). Additionally, flea infestation patterns show seasonal variation, with males more commonly infested (Gorrell and Schulte-Hostedde, 2008; Patterson *et al*., 2015). Given their ecological importance, red squirrels offer a valuable system for studying parasite community ecology, where parasite interactions could have implications for host fitness and broader ecological patterns.

Co-occurrence patterns of ectoparasites (two flea species and a chigger mite) on red squirrels were examined. This study evaluated (1) whether ectoparasites exhibit non-random co-occurrence patterns and (2) which, if any, host or external environmental factors influenced parasite assemblages. It was expected that any non-random co-occurrence patterns would be positive associations, as commonly seen in studies of ectoparasites on small mammal hosts (Krasnov *et al*., 2010; Nava and Lareschi, 2014; Colombo *et al*., 2015; but see Hoffmann *et al*., 2016; Veitch *et al*., 2020). It was also expected that parasite species co-occurrence patterns would be shaped by host and external environmental factors. Flea recruitment rates are often linked to the host's biology, while mite recruitment is more dependent on external environmental conditions (Linardi and Krasnov, 2013). If there are species associations between flea species, host traits will be more influential. If there are species associations between flea and mite species, either host traits or external environmental traits may play a role.

## Materials and methods

### Study area and field sampling

This study was conducted in Algonquin Provincial Park, Ontario, Canada (45°54′ N, 78°26′ W) from May to August of 2013 and 2015. North American red squirrels were captured in a 23-ha grid in an area of mixed deciduous-coniferous forest using Tomahawk live traps (Tomahawk Live Trap Co., Hazelhurst, Wisconsin, USA; detailed methods in Gorrell and Schulte-Hostedde, 2008). Tomahawk live traps were mounted 20-m apart at a height of approximately 1.5-m from the ground on platforms that were attached perpendicularly to randomly assigned mature trees that were large enough to attach and support the traps. Traps were padded with polyester stuffing and baited 06.00–19.00 h with an apple slice and a ~10-g mixture of peanut butter and oats, then checked in less than or equal to 2-h intervals.

Captured red squirrels were transferred to a handling bag, sexed, and assessed for reproductive status (non-reproductive or reproductively active). Squirrels were considered reproductively active if males were scrotal or females were lactating. Individuals were weighed using a Pesola® scale (±0.1 g) and received two metal ear tags with unique alphanumeric codes (National Band and Tag Co., Newport, Kentucky, USA). All methods used were reviewed and approved by the Animal Care Committee (ACC) at Laurentian University, Sudbury, Ontario, Canada, protocol number 2013-05-01.

### Collection of ectoparasite specimens

Ectoparasites were collected from squirrels during each capture with a metal flea comb (teeth spacing <300-*μ*m, one-tenth the size of the smallest fleas; Burgham Ltd., Toronto, Ontario, Canada) by combing ten times down the mid-back from the neck to the base of the tail, and ten times down the ventral surface from the sternum to the genitals. Ectoparasites collected were preserved in Eppendorf vials with 70% ethanol. Red squirrels in Canada are known to carry a variety of ectoparasites, including fleas (*Opisodasys pseudarctomys*, *Orchopeas caedens*, *Monopsyllus vison*, *Taropsylla coloradensis*), lice (*Hoplopleura sciuricola*, *Neohaematopinus sciurinus*), ticks (*Ixodes scapularis*, *Ixodes angustus*), and mites (Trombiculidae family; Gorrell and Schulte-Hostedde, 2008; Bouchard *et al*., 2011; Patterson *et al*., 2013; Bobbie *et al*., 2017). While combing is a reliable method for examining fleas, lice, and ticks, it is possible that mite species can be missed through visual inspection and combing (Beaumont *et al*., 2019); therefore, we may have been unable to identify additional mite species. However, this is a common method of quantifying ectoparasite communities on small mammals (e.g., Buchholz and Dick, 2017; Pero and Hare, 2018; Beaumont *et al*., 2019).

### Taxonomic identification of ectoparasite specimens

Fleas were sent to the Canadian Centre for DNA Barcoding (CCDB) at the University of Guelph, Ontario, Canada. A glass fibre protocol (Ivanova *et al*., 2006) was used to extract DNA from the macerated flea tissues; the 658-bp target region of the COI gene was amplified by polymerase chain reaction (PCR). Each 12.5-*μ*L PCR mixture included 6.25-*μ*L of 10% trehalose, 1.25-*μ*L 10× PCR buffer, 0.625-*μ*L (50-mm) MgCl2, 0.125-*μ*L (10-*μ*m) of each oligonucleotide primer, 0.0625-*μ*L (10-mm) dNTPs, 0.06-*μ*L Taq polymerase and 2-*μ*L ddH2O + 2-*μ*L template DNA (Hajibabaei *et al*., 2007). PCRs were run at the following thermal cycle conditions: 1-min at 94°C, followed by five cycles of 30-s at 94°C, 40-s at 50°C, and 1-min at 72°C, followed by 35 cycles of 30-s at 94°C, 40-s at 55°C, and 1-min at 72°C, and finally 10-min at 72°C. DNA extracts were PCR amplified using the forward and reverse primer-pair C_LepFolF (5′-ATTCAACCAATCATAAAGATATTGG-3′) and C_LepFolR (5′-TAAACTTCTGGATGTCCAAAAAATC-3′) respectively (Stein *et al*., 2013). PCR products were bidirectionally sequenced using Sanger sequencing with BigDye v3.1 using an ABI 3730 × l DNA Analyzer (Applied Biosystems, Foster City, CA).

The Refined Single Linkage (RESL) algorithm was used to cluster species (Ratnasingham and Hebert, 2013). Sequences and other pertinent specimen data (ex. date collected, its host, etc.) were uploaded to the Barcode of Life Data Systems (BOLD), and their OTUs were ascribed a Barcode Identification Number units (BINs); each BIN is populated by individual specimens having high sequence similarity and connectivity (Ratnasingham and Hebert, 2013). Nucleotide sequence homology searches were performed on the sequences obtained from the CCDB using NCBI BLAST (http://blast.ncbi.nlm.nih.gov/Blast.cgi).

Following sequence analysis and clustering, specimens were mounted on slides similar to the procedures laid out in Richards (1964). Maceration was completed by sequencing technicians at the CCDB. The exoskeletons were then transferred singly into 70% then 95% ethanol solutions for 3–5 min each for dehydration. Immediately after, they were placed overnight in oil of cloves. Specimens were mounted with Permount medium (Fisher Scientific) and placed in a drying oven at 50°C for a week. Further batches of slides were alternatively left to dry on the counter overnight. These mounts were imaged and mailed to Dr T. Galloway (University of Manitoba, Canada) for morphological identification. Dr Galloway was unaware of the DNA-barcoding results at the time of morphological identification, therefore providing a separate identification process to further support the DNA barcoding results. Flea morphological identification matched barcode species identification, apart from a single instance (which was excluded from the statistical analysis). One flea specimen failed the sequencing process and was only identified to species morphologically by Dr Galloway. The mite specimens did not undergo barcode species identification but were stored in 70% ethanol and mailed to Dr H. Proctor (University of Alberta, Canada) for morphological identification (Bobbie *et al*., 2017).

### Statistical analysis

Recaptures of individuals showed that fleas and mites take ~3–4 days to recolonize a host after parasite removal (data not shown). Therefore, recaptures of individuals that had ectoparasites removed were not included in the dataset unless at least a week had passed since ectoparasite removal. We only focused on occurrence in our statistical analysis, as parasite removal may influence intensity measures. Furthermore, only ectoparasites with at least 10 occurrences were included to avoid complications due to small sample sizes. This led to the exclusion of a single flea species (*Opisodasys pseudarctomys*). Ten flea specimens were missing information on the host's ID number or reproductive status and were also excluded from the statistical analysis. Analyses were conducted using statistical software package R version 4.0.2. Parasite prevalence, defined here as the proportion of host individuals infested with a parasite, was calculated by dividing the total number of infestation occurrences by the total number of host captures. Confidence intervals (95%) for parasite prevalence were calculated by Clopper-Pearson's exact method for binomial proportions (‘GenBinomApps’ package version 1.1). We ran linear mixed-effects models (‘nlme’ package version 3.1–149) with days since previous capture and Julian date as fixed effects and individual ID number as a random effect, but there was no effect of days since previous capture on occurrence of the flea species *Ceratophyllus vison* (*β* = 0.001, *P* = 0.674), *Orchopeas caedens* (*β* = −0.002, *P* = 0.315), or the mite *Neotrombicula harperi* (*β* < 0.001, *P* = 0.958; Pinheiro *et al*., 2012). Individuals with only a single capture were excluded from the linear mixed-effects models.

We fitted the model using the ‘boral’ package version 1.8.1 (Hui, 2016). This statistical method uses a model-based, parsimonious approach to ordination, with a generalized linear model and incorporated latent variables. Boral also accounts for host and external environmental factors while examining residual co-occurrence patterns (i.e. identification of species associations after controlling for investigated predictors). The model uses a species occurrence matrix as the response variable and host and external environmental covariates as explanatory predictors. We fitted a correlated response model, which examined how the parasite assemblage is explained by the host and external environment (Hui, 2016). Host sex and date were incorporated as fixed effects to describe the host and external environment. Date was centred and scaled by the mean and standard deviation. Host ID was included as a random effect.

We ran the Bayesian MCMC sampler in boral allowing for two latent variables with 200 000 iterations, the first 100 000 discarded as burn-in, and the remaining thinned by a factor of 100. The selection of two latent variables compromises between model complexity and appropriate evaluation of species co-occurrence patterns after controlling for host and external environmental predictors (Letten *et al*., 2015; Warton *et al*., 2015; Taranu *et al*., 2021). Random row effects were included to account for spatial variation. Including random row effects is equivalent to including a random intercept in mixed models, as a normal distribution is drawn with mean zero and unknown variance (Hui, 2016). The model was evaluated using stochastic search variable selection (SSVS) to determine predictors included in the final model (George and McCulloch, 1993). Only predictors with an SSVS mean >0.5 were included in the final model (sex: mean = 0.61, s.d. = 0.36; date: mean = 0.82, s.d. = 0.29). Predictors with an SSVS mean <0.5 were removed in a backwards stepwise format. This included the following predictors: host body mass, host reproductive status, host population abundance, and year removed based on SSVS values. Convergence was assessed using Dunn–Smyth residual and normal quantile residual plots and the Geweke diagnostic (Supplemental Information; Geweke, 1992). We identified the relative importance of host and external environmental factors for each parasite species and constructed a horizontal line plot showing 95% highest posterior density (HPD) intervals for the column-specific regression coefficients. We determined parasite species co-occurrences due to host and external environmental responses using the function ‘get.enviro.cor’ and the remaining co-occurrence patterns after controlling for predictors using the function ‘get.residual.cor’. To measure how well the predictors described the species assemblage, we calculated a proportional difference in the trace of the residual covariate matrix between the correlated response model and a pure latent variable model (where species are regressed against unknown covariates to produce an unconstrained ordination for visualizing site and species patterns; Warton *et al*., 2015; Hui, 2016). Plots of co-occurrence patterns were produced using the function ‘corrplot’. A horizontal line plot of the predictors and a plot for the estimates of variance partitions (Supplemental Information) were constructed using the functions ‘gg_coefsplot’ and ‘gg_varpart’ respectively from ‘ggboral’ package version 0.1.7.

## Results

The final dataset included 53 red squirrels (19 females, 34 males) with 207 captures, ranging from 1 to 19 captures for each individual. From DNA barcoding and morphological species identification, three prominent ectoparasite species were identified and analysed; two flea species (*C. vison* and *O. caedens*) and a chigger mite species (*N. harperi*) (Table 1). Eighty-four flea specimens were successfully sequenced and NCBI Blast identified two flea species with more than 95% identity with the known subtypes in GenBank (Table 2).

**Table 1.** Ectoparasite prevalence and number of red squirrels (*n* = 53 individuals, 207 captures) infested

Parasite species	Number of occurrences	Infested individuals	Prevalence (%, 95% CI)
Mite (*Neotrombicula harperi*)	11	8	05.31 (02.68–09.31)
Flea (*Orchopeas caedens*)	29	19	14.01 (09.59–19.50)
Flea (*Ceratophyllus vison*)	33	21	15.94 (11.24–21.65)
Flea (*Opisodasys pseudarctomys*)	1	1	00.48 (00.01–02.66)

Individuals captured multiple times that were infested by an ectoparasite species in at least one capture are included in the count of infested individuals.

**Table 2.** Highest identity coverage NCBI BLAST hit sequences from the COI gene for flea specimens collected from red squirrels (*n* = 84 sequences)

Accession number	Species identified	Range of percentage of shared identity (%)	Number of sequences
HM398797.1	*Ceratophyllus vison*	99.24–100.00	33
MG376790.1	*Ceratophyllus vison*	95.74–100.00	6
MG377835.1	*Ceratophyllus vison*	100.00	2
MG374778.1	*Ceratophyllus vison*	100.00	1
MG380123.1	*Ceratophyllus vison*	100.00	1
MG383124.1	*Ceratophyllus vison*	100.00	1
HM398830.1	*Orchopeas caedens*	98.77–99.83	34
KR140468.1	*Orchopeas caedens*	99.62–100.00	3
KR142021.1	*Orchopeas caedens*	100.00	1
KR146213.1	*Orchopeas caedens*	100.00	1
KR146262.1	*Orchopeas caedens*	100.00	1

**Figure 1.**
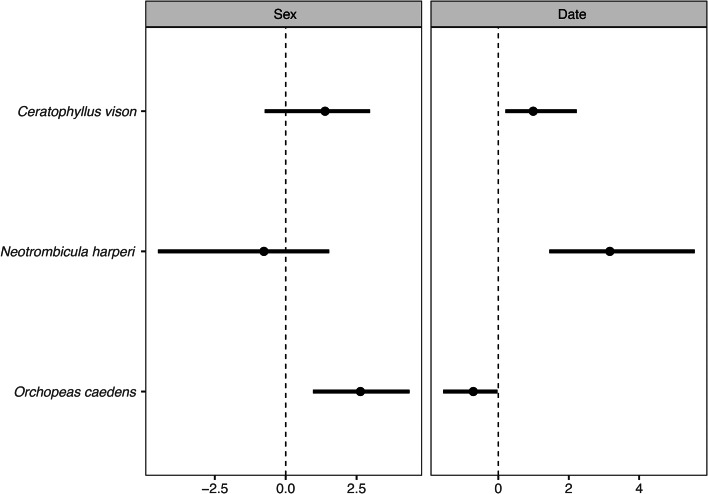
Effect estimates of host and external environment covariates as predictors of ectoparasite species occurrence. Circles represent posterior mean coefficients and horizontal lines represent 95% highest posterior density (HPD) intervals. Vertical dotted lines indicate the zero value.

There were no significant associations between parasite species after controlling for variation in occurrence with host sex and date. There was a negative association between the flea *O. caedens* and mite *N. harperi* and a positive association between the flea *C. vison* and *N. harperi* explained by host or external environmental responses (Table 3). Parasite species had varied responses to host sex and date (Fig. 1). *O. caedens* flea occurrence was greater on male than female hosts. All parasite species occurrences varied with date, where *C. vison* flea and *N. harperi* mite occurrences increased between spring and summer, and *O. caedens* flea occurrence decreased. Notably, the estimated residual covariate matrix decreased from 31.46 to 17.48 from a pure latent variable model to the correlated response model, suggesting that the investigated predictors explained only 13.98% of the co-occurrence patterns between parasites.

**Table 3.** Summary of correlations between ectoparasite species (fleas (*Orchopeas caedens* and *Ceratophyllus vison*) and mite (*Neotrombicula harperi*)) on red squirrels (*n* = 207 captures)

	Residual correlations		Correlations due to host and external environmental predictors	
	Estimate	95% HPD Intervals	Estimate	95% HPD Intervals
*Neotrombicula harperi* – *Orchopeas caedens*	0.288	−0.952–1.000	**−0.652**	**−1.000–(−0.205)**
*Neotrombicula harperi* – *Ceratophyllus vison*	−0.333	−1.000–0.907	**0.725**	**0.142–1.000**
*Orchopeas caedens* – *Ceratophyllus vison*	0.650	−0.405–1.000	0.069	−0.856–0.796

Bolded terms are those with 95% highest posterior density (HPD) intervals that do not include 0 (considered to have strong support).

## Discussion

A combination of positive, negative and random co-occurrence patterns was observed between the three prominent ectoparasite species on red squirrels. While we expected largely aggregative patterns, there were no significant associations between parasite species pairs when controlling for host sex and date. The significant associations observed, both positive and negative, were identified between the flea species and *N. harperi* mite and were explained by date. These trends suggest that associations between flea and mite species on red squirrels are shaped by the external environment rather than host traits or species interactions.

We did not identify any significant associations between parasite species pairs after controlling for host sex and date, indicating a lack of species interactions. Ectoparasite communities of small mammals tend to exhibit non-random co-occurrence patterns, particularly aggregation (Krasnov *et al*., 2010; Nava and Lareschi, 2014; Colombo *et al*., 2015), but this is not always the case (see Krasnov *et al*., 2006). Notably, Eurasian red squirrels (*Sciurus vulgaris*; Romeo *et al*., 2013) and invasive Pallas's squirrels (*Callosciurus erythraeus*; Mazzamuto *et al*., 2016) have hosted poor parasite assemblages with little variation in composition. Our investigated parasite community was also very small, which may provide little opportunity for transmission across hosts (Romeo *et al*., 2013). This suggests that the investigated ectoparasites of red squirrels are largely unstructured by interspecific competition or facilitative processes and follow what we would expect from stochastic processes.

While parasite occurrence patterns do not seem to be altered by parasite species interactions, significant co-occurrence patterns were identified in the correlated response model, suggesting that host and external environmental factors play a stronger role. *N. harperi* mites had a negative association with *O. caedens* fleas and a positive association with *C. vison* fleas. These co-occurrence patterns align with temporal trends in occurrence on red squirrel hosts that were also identified from our model (i.e. associations with date). Temporal trends in the external and host environment may play more of a role in whether parasite species pair co-occur more or less often than expected by chance. Ectoparasites have direct contact with the external environment, compared to endoparasites within the host's body (Bush *et al*., 2001), and consequently, co-occurrence patterns may be much more structured by these environmental conditions that change over time. However, it is important to note that the investigated predictors explained only ~14% of co-occurrence patterns between species and date may play a limited role in influencing the association between the investigated species pairs. This may also suggest that even weak associations of ectoparasite occurrence with date could structure co-occurrence patterns between species.

Notably, we only saw trends between *N. harperi* mites and both investigated flea species, but not between the two flea species. *N. harperi* mites had the strongest response to sampling date, which may explain this result. Temporal trends in the external environment, particularly ambient temperature and relative humidity, are well known to strongly impact the imago and pre-imago stages of mites and fleas, with downstream effects on survival, development time, and patterns of blood digestion (Linardi and Krasnov, 2013). Given that mite recruitment, compared to fleas, is often more tied to the external environment, we may expect greater variation in mite occurrence over time associated with changes in temperature and humidity (Linardi and Krasnov, 2013). This is especially true in Trombiculid mites, such as *N. harperi*, which are only parasitic in their larval stage and are often associated with particular soil or habitat characteristics rather than hosts (Timm, 1985). Therefore, the stronger ties that *N. harperi* shares with the external environment rather than the host environment may explain why date shaped associations between *N. harperi* and the two flea species, but not between the two flea species.

The negative trend of *O. caedens* flea occurrence over the sampling period was surprising, given that there was an increase in infection patterns over the same sampling period in the study area 10 years prior (Gorrell and Schulte-Hostedde, 2008). However, a similar negative trend was seen over the same seasonal sampling period in eastern chipmunks (Amin, 1976) for a flea in the same genus, *Orchopeas h. howardii*. Moderate differences between years in seasonal trends of *Orchopeas sexdantatus* flea infestations on desert woodrats have also been observed, with occurrence patterns varying with humidity (Lang, 2014), suggesting that we may expect differences in occurrence patterns of our investigated flea species between different years of study. Temporal temperature trends can shape flea occurrence patterns through impacts on flea oviposition, egg clutch size, immature development and survival (Bossard, 2022). Thus, we may expect changes in *O. caedens* flea occurrence throughout our study.

*N. harperi* mite and *C. vison* flea occurrence increased over the sampling period. This was expected, as previous studies have noted that both of these parasite groups tend to exhibit seasonal patterns where they increase during the warmer months. *N. harperi* mites tend to emerge in May or June and reach higher occurrences in the summer (Brennan and Wharton, 1950). Our sampling period falls within periods where infestation rates should be increasing, as chigger mites (such as *N. harperi*) are only active during warm months in northern temperate areas (Dietsch, 2005). Additionally, fleas in the *Ceratophyllus* genus often exhibit peaks during summer months (Samurov, 1990; Cyprich and Krumpal, 2001; Haukisalmi and Hanski, 2007). The results of our study are consistent with previous work on *N. harperi* and *C. vison* on mammalian hosts.

Approximately 14% of the variation in co-occurrence patterns between the investigated parasite species was explained by our predictors, indicating that there may be additional factors that play a more substantial role in shaping parasite communities in this system. While we did investigate date, particular seasonal conditions such as temperature, rainfall, and humidity can influence flea occurrence on squirrels (Goldberg *et al.*, 2020; Smith *et al*., 2021). Flea species can have similar or differing physiological tolerances to environmental conditions, which can shape their occurrence patterns (Smith *et al*., 2021). Precipitation levels from the previous year can have a strong effect on flea abundance on squirrels (Goldberg *et al*., 2020). For chigger mites of small mammals, occurrence may vary with habitat type (Choi *et al*., 2019; Matthee *et al*., 2020). The host community within each habitat can also influence the occurrence of chigger mites, as additional common host species may facilitate greater mite abundance (Matthee *et al*., 2020). The abundance of host species that spend more time on the ground near these soil-dwelling mites, such as voles, may provide food resources to support the mite population (Veitch, 2020). Therefore, further investigation of environmental conditions, such as temperature, precipitation, humidity, habitat type, and the host community may provide better explanatory predictors of parasite co-occurrence on red squirrels.

The occurrence of *O. caedens* fleas was greater on male red squirrels. Male host bias is a common trend in fleas parasitizing rodents (Perez-Orella and Schulte-Hostedde, 2005; Krasnov *et al*., 2012) and has been observed in other study systems of red squirrels (Patterson *et al*., 2015). However, a female-bias in flea parasitism was previously identified in this host population (Gorrell and Schulte-Hostedde, 2008). Inspection of the raw dataset from Gorrell and Schulte-Hostedde (2008) demonstrated that there was higher flea parasitism on males recorded in June and August, suggesting that higher flea occurrence on male red squirrels is not uncommon in this population. Male squirrels often maximize number of matings with associated costs to their body condition and immune function (Scantlebury *et al*., 2010). Higher testosterone levels in males can lead to immunosuppressive effects (Folstad and Karter, 1992; Zuk and McKean, 1996; Foo *et al*., 2017; but see Rolff, 2002). Males also often engage in behaviours that increase encounter rates such as increased mobility in larger or overlapping home ranges, which increases contact between hosts and the likelihood of potential transmission (Krasnov *et al*., 2012). Male biases in *O. caedens* flea parasitism could be related to fitness benefits to the parasites, as fleas may feed more effectively and produce more offspring on male hosts (Khokhlova *et al*., 2009). Therefore, male red squirrels may be more susceptible to infestation of fleas. However, given that the investigated predictors explained ~14% of co-occurrence patterns between parasite species, further experimentation is needed to examine variation in flea occurrence with host sex.

The ectoparasite community of red squirrels investigated in this study did not appear to be structured by parasite-parasite species interactions but largely by changes in infestation patterns over our sampling period. External environmental conditions that fluctuate over time, such as temperature, rainfall, and humidity could play strong roles in structuring ectoparasite infestations and the impact of these factors on ectoparasite communities should be further explored. Ectoparasite communities of red squirrels may be shaped by seasonal or temporal shifts in the physical environment, but the host and external environmental factors that shape these associations are yet to be fully identified.

## Supporting information

Veitch et al. supplementary material Veitch et al. supplementary material

## Data Availability

Sequences, images, and original trace files are available for viewing in the project ‘Ectoparasites from Squirrels in Algonquin Park’ (ESAP), on the Barcode of Life Data Systems (http://www.boldsystems.org/).

## Supplementary material

The supplementary material for this article can be found at https://doi.org/10.1017/S0031182024001513.
