# Supplementary material for: Parasite species co-occurrence patterns on North American red squirrels (Tamiasciurus hudsonicus)
Source: Parasitology. 2024 Nov 18;151(14):1597–603. doi: 10.1017/S0031182024001513 (PMC12052431; doi:10.1017/S0031182024001513)
Supplement: Veitch et al. supplementary material [file S0031182024001513sup001.docx]

**Parasite species co-occurrence patterns on North American red squirrels (*Tamiasciurus hudsonicus*)**

**Authors:** Jasmine S. M. Veitch, Jeff Bowman, J. Dawson Ketchen, Albrecht I. Schulte-Hostedde

**SUPPLEMENTAL INFORMATION**

**Supplemental Figure 1.** Diagnostic plots for residual analysis of *Ceratophyllus vison* (red circles), *Neotrombicula harperi* (green circles), and *Orchopeas caedens* (blue circles). Plots are Dunn-Smyth residuals vs. linear predictions (A), Dunn-Smyth residuals vs. sites/hosts (B), Dunn-Smyth residuals vs. parasite species (C), and normal quantile plot of Dunn-Smyth residuals (D), respectively. Geweke diagnostic p = 0.958.


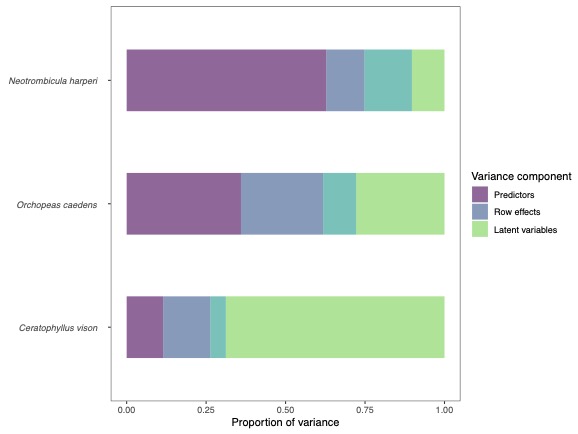


**Supplemental Figure 2.** Results of variance partitioning for boral model. Variation of parasite occurrence explained by predictors (i.e. host and external environmental covariates) and latent variables (i.e. species associations) for each parasite species of red squirrels (n = 53 individuals, 207 captures).
